# 16S rRNA gene sequencing reveals bacterial diversity in Khewra Salt Mine walls

**DOI:** 10.1099/acmi.0.000869.v4

**Published:** 2024-12-18

**Authors:** Syeda Wardah Noor, Sara Arshad, Syed Muhammad Abdullah, Bushra Khudadad Kayani, Sahar Fazal

**Affiliations:** 1 Department of Bioinformatics and Biosciences, Capital University of Science and Technology, Islamabad, Pakistan; 2 Department of Science and Environment, Griffith University, Brisbane, Queensland, Australia

**Keywords:** 16s rRNA sequencing, *Bacillus*, *Halobacteriaceae*, halophile, Khewra Salt Mine, salt, salt tolerance

## Abstract

The Khewra Salt Mine, also known as the source of Himalayan Pink Salt, is the second largest salt mine in the world and a significant site for salt export. The mine’s salt exhibits both amorphous and crystalline textures, with colours ranging from white to deep red, contributing to its distinctive pink hue. While previous research has extensively explored the mine’s soil, plants and brine samples, little attention has been given to the salt walls, which are a major tourist attraction. This study aimed to investigate the bacterial diversity within the salt walls from four distinct locations, including an under-construction area sampled for the first time. From these four collection points, 7 samples were gathered, yielding 19 isolates cultured on 2% NaCl nutrient agar. Bacterial isolates from the under-construction area (US1-1, US1-2, US2-1 and US2-2) and the museum walls (MS1-1 and MS1-2) underwent further biochemical, antibiotic sensitivity and salt requirement testing, followed by 16S rRNA sequencing. Biochemical assays identified these isolates as Gram-positive, catalase- and oxidase-positive rods. All isolates were sensitive to chloramphenicol (30 µg ml^−1^). Salt tolerance tests classified them as slightly halophilic, thriving at 2–4% NaCl concentrations. Molecular characterization revealed two strains from the under-construction area, US1-1 and US2-2, as uncultured *Bacillus* species clone 5 and *Bacillus* sp. PSA3, respectively, both with 94% sequence similarity. The phylogenetic results show monophyletic associations between these two strains, which could be due to their shared salt location and common ancestors. The sequences of US1-2, US2-1 and MS1-1 were 100% identical to *Bacillus licheniformis* strain P8_B2 chromosome, while MS1-2 matched *Staphylococcus saprophyticus* strain CJJH1 with 100% similarity. The results of this study point towards a significant diversity of bacteria, slightly halophilic, cocci or bacillus-shaped. These halophilic bacterial strains, possessing salt-tolerant genes, present the need for further identification of bacterial colonies in the mine and their evolutionary patterns, which could also help uncover the role these microbes play in the ecosystem of the salt mine. Additionally, they show promise for industrial enzyme production.

**Impact Statement**

This study has unveiled the diverse bacterial species inhabiting the salt walls of Khewra, focusing particularly on halophiles and their required salt concentrations. By identifying 19 isolates predominantly from the *Bacillus* genus, which thrive in 2–4% NaCl concentration, this research fills a significant knowledge gap. The findings highlight the importance of these bacterial strains in salt walls of the newly mined regions of the salt mine, setting the stage for further investigation into their evolutionary traits and ecological roles. In the context of Pakistan’s agriculture, these halophilic *Bacillus* strains could be pivotal in developing transgenic salt-tolerant crops, thereby improving productivity in saline soils. Additionally, given *Bacillus* species established industrial applications in enzyme production, antibiotic synthesis, biofuel generation and bioremediation, the results underscore their potential economic and environmental benefits. This research not only enhances our understanding of bacterial life in extreme environments but also presents practical implications for biotechnology and sustainable agriculture.

## Data Summary

16S rRNA sequences submitted in GenBank: US1-1 (accession code OQ295687), US1-2 (accession code OQ978562), US2-1 (accession code OQ978592), US2-2 (accession code OQ692139), MS1-1 (accession code OQ978561) and MS1-2 (accession code OQ978593).

## Introduction

Salt is an ionic compound that drives major industries as a preservative and antimicrobial agent in the food industry [1], feedstock in the chemical industry [2] and in medicine [3]. The salt mines and salt lakes are the source of multiple salts, characterized based on their constitutive elements such as magnesium, sodium, potassium and sulphur. Salt intake regulates body fluid levels and facilitates nerve impulse transmission, muscle contraction, nutrient availability and transport across cells. However, excessive consumption may result in hypertension and kidney and cardiovascular disorders. Traditional and modern salt mining techniques are employed to excavate salt from deep salt caves and oceans [4]. Currently, the world’s top reported salt mines are Sifto Salt Mine (Canada), Khewra Salt Mine (Pakistan) and Prahova Salt Mine (Romania), respectively.

Salt mines inhabit a diverse microbiome predominantly halophiles or salt-loving micro-organisms categorized under extremophiles thriving in extreme conditions. Evolutionary mechanisms have enabled halophiles to tolerate high salt concentrations by extruding harmful inorganic ions and accumulating necessary solutes, salt-resistant proteins and amino acids often through a membrane-bound proton pump known as bacteriorhodopsin [5,6]. Halophiles from *Archaea*, *Bacteria* and *Eukarya* inhabit lands, sea and rocks. According to Roohi *et al*. [7], bacteria that survive in 1–5% NaCl concentration are slightly halophilic, 5–15% NaCl concentration is for moderately halophilic, and lastly, extremely halophilic requires 15–40% NaCl to grow. The *Halobacteriaceae* family is the largest with 36 genera and 129 salt-dependent species that include Gram-negative halophilic *Marinomonas*, *Alteromonas*, *Acinetobacter*, *Vibrio* and *Pseudomonas* [8]. Likewise, organisms of the genera *Bacillus*, *Salinococcus*, *Marinococcus* and *Sporosarcina* have been found thriving in slatterns and salted soils [9].

Khewra Salt Mine (32.6480° N, 73.0084° E) is the world’s second largest salt mine, located in Pind Dadan Khan Tehsil of Jhelum District, Punjab, Pakistan. It is the key player in Pakistan’s salt export, currently operating under the authority of Pakistan Mineral Development Corporation (PMDC), generating up to 314 845 tons of salt per annum [10]. The trade value from the export of Pakistani salt and salt products sums up to 59.65M USD for the year 2020 [11]. The mine is open to the public harnessing a museum illuminated with salt sculptures and brine ponds. The salt in Khewra comprises sodium chloride, potassium chloride and calcium sulphate mostly in transparent white, greyish black, reddish brown and pink hues like the Himalayan salt known worldwide [12]. PMDC has also concocted an asthma resort in Khewra Mines, laced with 10 beds and 50 seats to offer halotherapy for the allergic ones, following Polish and Ukrainian salt mines [10]. Halotherapy is the inhalation of tiny salt particles in timed sessions to treat respiratory disorders and allergies. It clears respiratory pathways, reduces oxidative damage, boosts immunity and facilitates skin regeneration [13].

Over the past few years, research in Khewra has focused on its halophytes through the identification of bacterial strains including *Salsola stocksii*, *Atriplex amnicola*, *Bacillus megaterium, Staphylococcus pasteuri, Pseudomonas aeruginosa* and *Pseudomonas putida* [14,15]. *Bacillus anthracis* and *Staphylococcus saprophyticus* have been identified in its saline water [16].

The primary goal of this research was to investigate the bacterial diversity within the salt walls of Khewra Mine, utilizing 16S rRNA sequencing technology. The results were promising, with 19 bacterial colonies successfully isolated from 7 salt samples. Molecular characterization of six isolates using 16S rRNA sequencing identified two strains, US1-1 and US2-2, from the working area as uncultured *Bacillus* sp. clone 5 and *Bacillus* sp. PSA3, both showing 94% similarity. Additionally, three sequences, US1-2, US2-1 and MS1-1, were found to be 100% identical to *Bacillus licheniformis* strain P8_B2 chromosome, while MS1-2 matched 100% with *S. saprophyticus* strain CJJH1. These findings, supported by morphological and biochemical analyses, highlight the significant potential for bacterial diversity in the Khewra Salt Mine.

## Methods

### Description of sample location

**Fig. 1.**
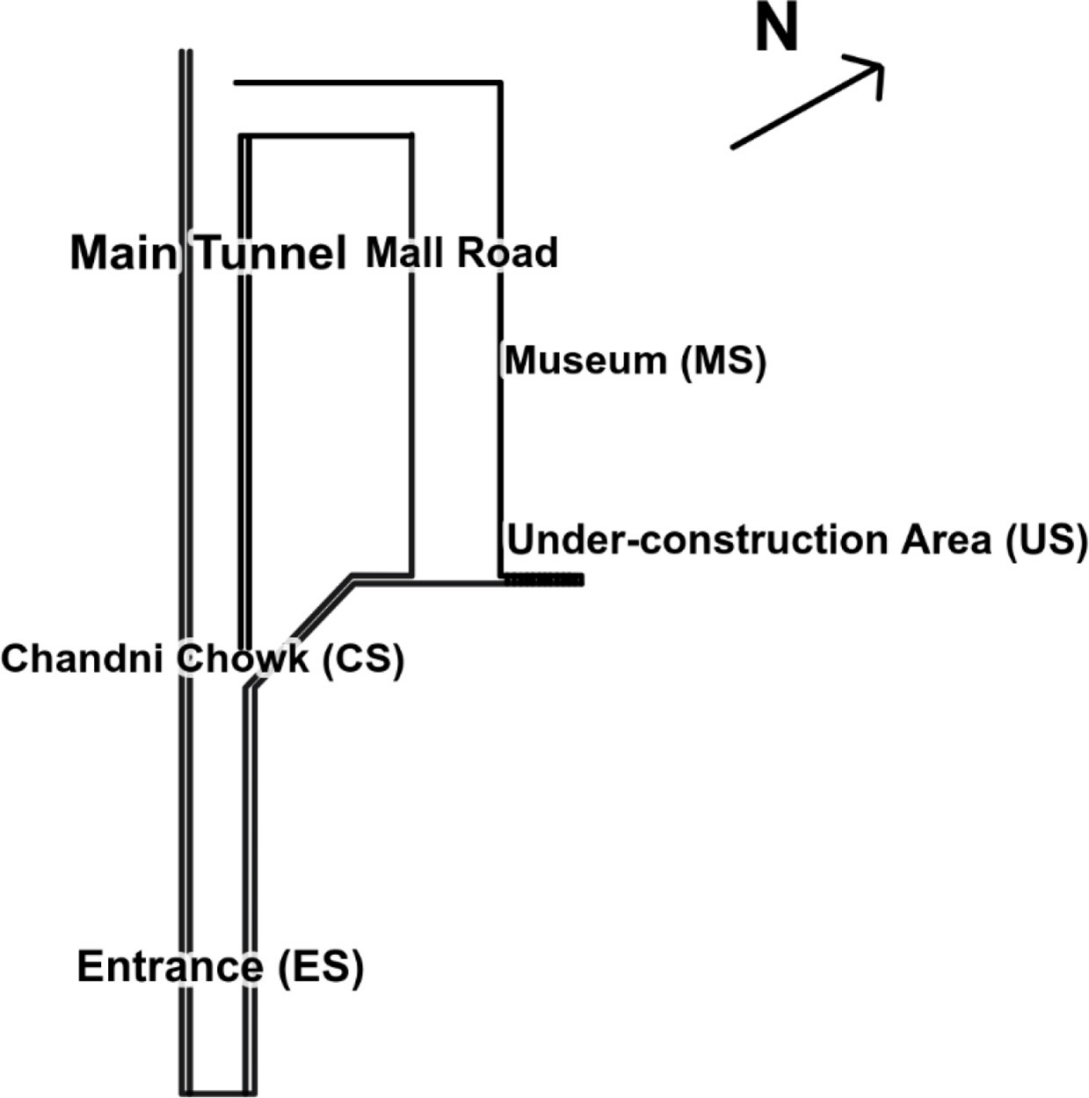
Sketch map shows the general location points of samples discussed in paper.

The main entrance tunnel at ground level in Khewra Salt Mine leads to a centre point, Chandni Chowk, which yields two passageways. Moving right from Chandni Chowk, there is a short lane of brine ponds that ends at two other passages; on the right is the museum, and on the left is the under-construction tunnel, an area subject to active mining and not open to the public. The museum starts with a small mosque and has many salt sculptures. The museum ends with the main tunnel, which leads back to Chandni Chowk and entrance site. The north-west tunnel leads to ponds, namely Shish Mahal and Brine Pond. A simple sketch map has been shown in Fig. 1 for elaboration of sample locations only.

### Collection of salt samples

A total of seven salt samples were collected from four different locations within the salt mine varying in colour and texture. The sample locations in Table 1 were categorized as entrance of mine (ES) roughly 150 m into the main tunnel, area preceding Chandni Chowk (CS), museum area roughly 800 m towards the end of mine (MS-1, MS-2 and MS-3) and under-construction area of salt mine (US-1 and US-2). The samples were packaged separately in zip-locked bags and transported in cool boxes to the laboratory.

**Table 1.** Location of salt samples, their sample names and codes

Location	Salt sample name	Salt sample codes
Entrance of mine	Entrance salt	ES
Chandni Chowk	Chandni salt	CS
Museum, Mall Road	Museum salt	MS
Under-construction area	Under-construction salt	US

### Sample preparation

The salt colour and texture were observed using the naked eye under bright light. The pH of samples was recorded through an electronic pH metre. The salt samples were crushed with a sterile pestle and mortar and processed by adding 2 g of crushed salt in 5 ml water.

### Isolation of bacteria and morphological characterization

A 0.5 ml of each sample dilution was spread on nutrient agar medium (0.5% NaCl) and incubated at 37 °C for 4 days, and plates with bacterial colonies ranging from 30 to 300 c.f.u. were selected. Each inoculated plate was recorded for bacterial growth based on colony morphology such as margins, elevation, form, colour and opacity using the naked eye. These distinctive bacteria were further streaked on 2% NaCl nutrient agar and incubated at 37 °C for 24 h to obtain their pure cultures. Gram staining [17] was performed to identify bacteria based on their cell wall composition.

### Biochemical characterization of bacterial isolates

Biochemical characterization of 19 isolated bacterial strains was performed through catalase test [18], oxidase test [19] and carbohydrate fermentation test [20]. For US1-1, US1-2, US2-1, US2-2, MS1-1 and MS1-2 strains, Simmon citrate [21] and sulfide indole motility (SIM) tests [22] were performed for further biochemical characterization.

### Molecular characterization of bacterial isolates

For the molecular characterization of samples, the isolates US1-1, US1-2, US2-1, US2-2, MS1-1 and MS1-2 were selected based on sample location, colony morphology and biochemical results. These six bacterial isolates were inoculated on nutrient agar and incubated for 24 h at 37 °C to obtain pure isolates.

#### DNA extraction

The genomic DNA of six isolates was extracted using cetyl trimethylammonium bromide [23] with slight modifications to optimize the quality of DNA.

#### PCR amplification

The extracted DNA was used as a template for 16S rRNA PCR (Bio-Rad T100 PCR Thermal) using 341F (5′-CCTAYGGGRBGCASCAG-3′) 17mer and 806R (5′- GGACTACNNGGGTATCTAAT-3′) 20mer. The DNA extracted from US1-2, US2-1 and MS1-1 failed to yield specific results with these primers. Therefore, they were amplified again with 16S primers, 9F (5′-GAGTTTGATCCTGGCTCAG-3′) 19mer and 1510R (5′GGCTACCTTGTTACGA-3′) 16mer. These amplified sequences were sent to Macrogen commercial service (Macrogen, Korea) for 16S rRNA sequencing.

### Salt requirement for the growth of pure isolates

Six purified bacterial isolates, US1-1, US1-2, US2-1, US2-2, MS1-1 and MS1-2, were selected to understand the salt requirements for growth on three different salt concentrations. They were grown on 2, 4 and 6% NaCl concentrations in nutrient agar [24]. The isolates were inoculated on media with varying salt concentrations and incubated at 37 °C, and the results were recorded the next day.

### Antibiotic sensitivity of isolates

Antibiotic sensitivity tests were performed for US1-1, US1-2, US2-1, US2-2, MS1-1 and MS1-2 using disc diffusion method [25]. Penicillin (10 µg ml^−1^), ampicillin (10 µg ml^−1^), chloramphenicol (30 µg ml^−1^) and erythromycin (15 µg ml^−1^) discs were placed on the bacterial plates which were then incubated at 37 °C for 24 h. The results were identified by measuring the zones of inhibition as sensitive (S), resistant (R) and intermediate (I) using the Kirby Bauer Chart.

#### Statistical analysis

The antibiotic sensitivity test results were analysed by one-way ANOVA, Tukey’s multiple comparisons and pairwise *t*-test using the ANOVA calculator [26].

### Phylogenetic analysis

For the assembly of 16S rRNA sequences, BioEdit software was used. The sequences were identified through blast tool on NCBI (National Center for Biotechnology Information), which helped narrow down the closely related species. The phylogenetic tree was constructed using mega X tool (version 11). *Escherichia coli* was used as outgroup, and six other strains of *Bacillus* sp. were used as ingroup alongside extracted six strains. The 16S rRNA sequences were downloaded in FASTA format. First, the sequences were aligned using clustalw and then assessed by the neighbour-joining (NJ) algorithm. By performing a bootstrap analysis with 1000 re-samplings of the NJ data for the tree topology, the relationship stability was assessed. All six sequences, US1-1, US1-2, US2-1, US2-2, MS1-1 and MS1-2, were submitted in NCBI GenBank with accession codes OQ295687, OQ978592, OQ978561, OQ692139, OQ978562 and OQ978593, respectively.

## Results

### Physical properties of salt samples

A total of seven salt samples with varying characteristics from the salt walls have been recorded in Table 2. The appearance of samples collected from all four sites was mostly crystalline with colours ranging from greyish and black to white and peach red.

**Table 2.** Physical characteristics of salt samples and their pH values in distilled water

Sample	Location	Colour	Texture	pH
***ES***	Entrance tunnel	Greyish brown	Fine crystals	7.03
***CS***	Chandni Chowk	White	Fine crystals	7.02
***MS1***	Museum salt wall 1	Ash grey with reddish brown crystals	Fine crystals	7.03
***MS2***	Museum salt wall 2	Reddish brown with black lumps	Fine crystals	7.02
***MS3***	Museum salt wall 3	Grey	Fine crystals	7.03
***US1***	Under-construction salt wall 1	Peach salt with black hues	Large, non-uniform crystals	7.03
***US2***	Under-construction salt wall 2	Salmon salt with red hues	Large, non-uniform crystals	7.03

### Macroscopic and microscopic characteristics of isolates

All the isolates formed colonies on 2% NaCl nutrient agar except ES-1. A total of 19 pure bacterial isolates were obtained from the four different locations of salt mine as shown in Table 3. Based on Gram staining, 17 out of 19 isolates were Gram-positive, and two were Gram-negative. Colony morphology revealed nine isolates as creamy white, four creamy yellow, two white, two yellow, one orange and one light yellow in colour, mostly opaque, circular and filamentous in appearance and cocci in shape.

**Table 3.** Macroscopic and microscopic features of isolates

Colony	Colour	Elevation	Margin	Form	Opacity	Gram stain	Cell shape
**ES-2**	Creamy white	Raised	Filiform	Filamentous	Opaque	+ve	Cocci
**ES-3**	Light yellow	Raised	Entire	Circular	Opaque	+ve	Cluster of cocci
**ES-4**	White	Raised	Filiform	Filamentous	Opaque	+ve	Diplococci
**CS-1**	Creamy yellow	Flat	Lobate	Filamentous	Translucent	+ve	Coccobacilli/coccoid
**CS-2**	Creamy white	Umbonate	Entire	Circular	Opaque	+ve	Cocci
**CS-3**	Creamy white	Flat	Undulate	Spread growth	Opaque	+ve	Cocci
**CS-4**	Creamy yellow	Umbonate	Entire	Circular	Opaque	−ve	Cluster of cocci
**MS1-1**	Yellow	Raised	Entire	Circular	Opaque	+ve	Rods
**MS1-2**	Orange	Raised	Entire	Circular	Opaque	+ve	Chain of rods
**MS1-3**	Creamy white	Raised	Entire	Circular	Opaque	+ve	Cocci
**MS1-4**	Creamy white	Flat	Undulate	Irregular	Opaque	+ve	Diplococci
**MS2-1**	Creamy white	Flat	Undulate	Irregular	Translucent	+ve	Cluster of cocci
**MS2-2**	Creamy white	Raised	Entire	Circular	Opaque	−ve	Cocci and cocci chains
**MS3-1**	Yellow	Raised	Entire	Circular	Opaque	+ve	Diplococci
**MS3-2**	White	Raised	Entire	Circular	Opaque	+ve	Diplococci
**US1-1**	Creamy yellow	Raised	Undulate	Rhizoid	Translucent	+ve	Rods
**US1-2**	Creamy yellow	Flat	Entire	Circular	Translucent	+ve	Rods
**US2-1**	Creamy white	Raised	Undulate	Filiform	Opaque	+ve	Coccobacillus/coccoid
**US2-2**	Creamy white	Raised	Undulate	Irregular	Opaque	+ve	Rods

**Table 4.** Results of biochemical test on all 19 isolates

Sample code	Catalase test	Oxidase test	Carbohydrate fermentation	
			Glucose	Sucrose
**ES-2**	+	−	+	+
**ES-3**	+	−	+	+
**ES-4**	+	−	+	+
**CS-1**	+	+	+	+
**CS-2**	−	+	+	+
**CS-3**	+	+	+	+
**CS-4**	+	+	+	+
**MS1-1**	+	+	+	+
**MS1-2**	+	+	+	+
**MS1-3**	+	−	+	+
**MS1-4**	+	−	+	+
**MS2-1**	+	−	+	+
**MS2-2**	+	+	+	+
**MS3-1**	+	+	+	+
**MS3-2**	+	−	+	+
**US1-1**	+	+	+	+
**US1-2**	+	+	+	+
**US2-1**	+	+	+	+
**US2-2**	+	+	+	+

+, Positive reaction; −, negative reaction.

**Table 5.** Results of biochemical test of selected six isolates for further biochemical properties

Sample	Citrate test	SIM test		
		**Sulfide**	Indole	Motility
**US1-1**	−	−	+	+
**US1-2**	+	−	+	+
**US2-1**	+	−	+	+
**US2-2**	−	−	+	+
**MS1-1**	+	−	+	−
**MS1-2**	−	−	+	+

+, Positive reaction; −, negative reaction.

Biochemical characterization revealed that most isolates were positive for catalase (18), oxidase (12) and carbohydrate (glucose and sucrose) fermentation test (Table 4).

Table 5 shows the separate biochemical tests performed for US1-1, US1-2, US2-1, US2-2, MS1-1 and MS1-2. All six isolates tested positive for indole and negative for sulfide. The citrate test was positive for three isolates. The motility test was negative for the MS1-1 isolate only.

### Salt requirement of pure isolates

The salt requirement for the growth of the isolated strains was determined by growing at different salt concentrations. According to the results in Table 6, salt concentration tests revealed significant growth overnight at 2 and 4% salt concentration but failed to grow on 6% indicating that all six isolates were slight halophiles [7].

**Table 6.** Salt requirement of selected six isolates using three different concentrations of NaCl in nutrient agar

Sample code	Growth in		
	2% NaCl (g l^−1^)	4% NaCl (g l^−1^)	6% NaCl (g l^−1^)
**US1-1**	+	+	−
**US1-2**	+	+	−
**US2-1**	+	+	−
**US2-2**	+	+	−
**MS1-1**	+	+	−
**MS1-2**	+	+	−

+, Positive reaction; −, negative reaction.

### Antibiotic sensitivity tests of selected isolates

The selected isolates were subjected to antibiotic sensitivity testing, which revealed that US1-1 was the most sensitive to all antibiotics and US1-2 had the most resistance to all antibiotics. Moreover, all isolates showed significant sensitivity to chloramphenicol (30 µg ml^−1^) (Table 7).

**Table 7.** Antibiotic sensitivity testing with penicillin (10 µg ml^−1^), ampicillin (10 µg ml^−1^), chloramphenicol (30 µg ml^−1^) and erythromycin (15 µg ml^−1^)

Sample	Antibiotics and their relative zones of inhibition			
	Erythromycin (15 µg ml^−1^)	Ampicillin (10 µg ml^−1^)	Penicillin (10 µg ml^−1^)	Chloramphenicol (30 µg ml^−1^)
**US1-1**	30 mm (S) 29 mm (S) 30 mm (S)	20 mm (S) 21 mm (S) 19 mm(S)	25 mm (R) 23 mm (R) 25 mm (R)	30 mm (S) 31 mm (S) 30 mm (S)
**US1-2**	6 mm (R) 7 mm (R) 6 mm (R)	No Zone (R) No Zone (R) No Zone (R)	11 mm (R) 9 mm (R) 11 mm (R)	22 mm (S) 20 mm (S) 21 mm (S)
**US2-1**	26 mm (S) 27 mm (S) 25 mm (S)	10 mm (R) 8 mm (R) 10 mm (R)	30 mm (S) 30 mm (S) 29 mm (S)	30 mm (S) 28 mm (S) 28 mm (S)
**US2-2**	30 mm (S) 28 mm (S) 29 mm (S)	No Zone (R) No Zone (R) No Zone (R)	8 mm (R) 8 mm (R) 9 mm (R)	30 mm (S) 29 mm (S) 30 mm (S)
**MS1-1**	30 mm(S) 29 mm (S) 29 mm (S)	10 mm (R) 11 mm (R) 10 mm (R)	10 mm (R) 10 mm (R) 10 mm (R)	30 mm (S) 29 mm (S) 29 mm (S)
**MS1-2**	10 mm(R) 10 mm(R) 8 mm(R)	10 mm (R) 9 mm (R) 9 mm (R)	35 mm (S) 34 mm (S) 36 mm (S)	30 mm (S) 31 mm (S) 30 mm (S)

#### Statistical analysis

The findings of ANOVA, Tukey’s multiple comparison and pairwise *t*-test of antibiotic tests for the control and bacterial isolates using the ANOVA calculator are summarized in Tables S1, S2 and S3, respectively, available in the online Supplementary Material.

The statistical analysis of antibiotic test results yields the *f*-ratio value of 39.66663 and the *P*-value of <0.00001. The *P*-value of <0.05 indicates a significant variation in drug susceptibility in the tested strains. The high *f*-value at 39.66663 shows that there is a statistically significant difference between the group means and the small *P*-value (1.0447e-18). The Tukey’s multiple comparison test, also known as the *post-hoc* test, was used to locate the significant differences between specific pairs of groups. All the strains tested against erythromycin (*P-*value 2.1357e-10), penicillin (*P*-value 1.2851e-8) and chloramphenicol (*P*-value 2.0838e-10) revealed significant differences as compared with the control and were found to be highly susceptible, whereas all of them appeared highly resistant against ampicillin with no significant difference (*P*-value 0.3309).

Comparisons using pairwise *t*-test between groups show very small *P*-values (e.g. 1.7281e−12 for control vs. erythromycin), indicating significant differences between the groups, except in a few cases where *P*-values are higher, showing no significant differences (e.g. *P*-value 0.0931 for erythromycin vs. penicillin). Overall, the ANOVA and *post-hoc* tests demonstrate that there are significant differences between the groups, particularly between the control group and most of the antibiotics (except for ampicillin), as well as between specific pairs of antibiotics.

### Molecular characterization and phylogenetic analysis of isolates through 16S rRNA sequencing

Table 8 details the closest known relatives of the six isolated strains through blast results from NCBI, their GenBank accession codes and percentage similarity. The sequences were submitted in NCBI GenBank, and their respective accession codes have also been listed in Table 8.

**Table 8.** Results of 16S rRNA describe the closest similarity of strains through blast as well as accession codes after submission in GenBank

Sample	Location of sample	Nearest strain (through blast)	Accession code of nearest strain	Accession code of strains after submission in GenBank	Percentage similarity
US1-1	Under-construction area (Fig. 4f)	Uncultured *Bacillus* sp. clone 5	DQ887535.1	OQ295687	94
US1-2	Under-construction area (Fig. 4f)	*B. licheniformis* strain P8_B2 chromosome	CP045814.1	OQ978562	100
US2-1	Under-construction area (Fig. 4g)	*B. licheniformis* strain P8_B2 chromosome	CP045814.1	OQ978592	100
US2-2	Under-construction area (Fig. 4g)	*Bacillus* sp. PSA3	KR401264.1	OQ692139	94
MS1-1	Museum wall (Fig. 3c)	*B. licheniformis* strain P8_B2 chromosome	CP045814.1	OQ978561	100
MS1-2	Museum wall (Fig. 3c)	*S. saprophyticus* strain CJJH1	EU169536.1	OQ978593	100

**Fig. 2.**
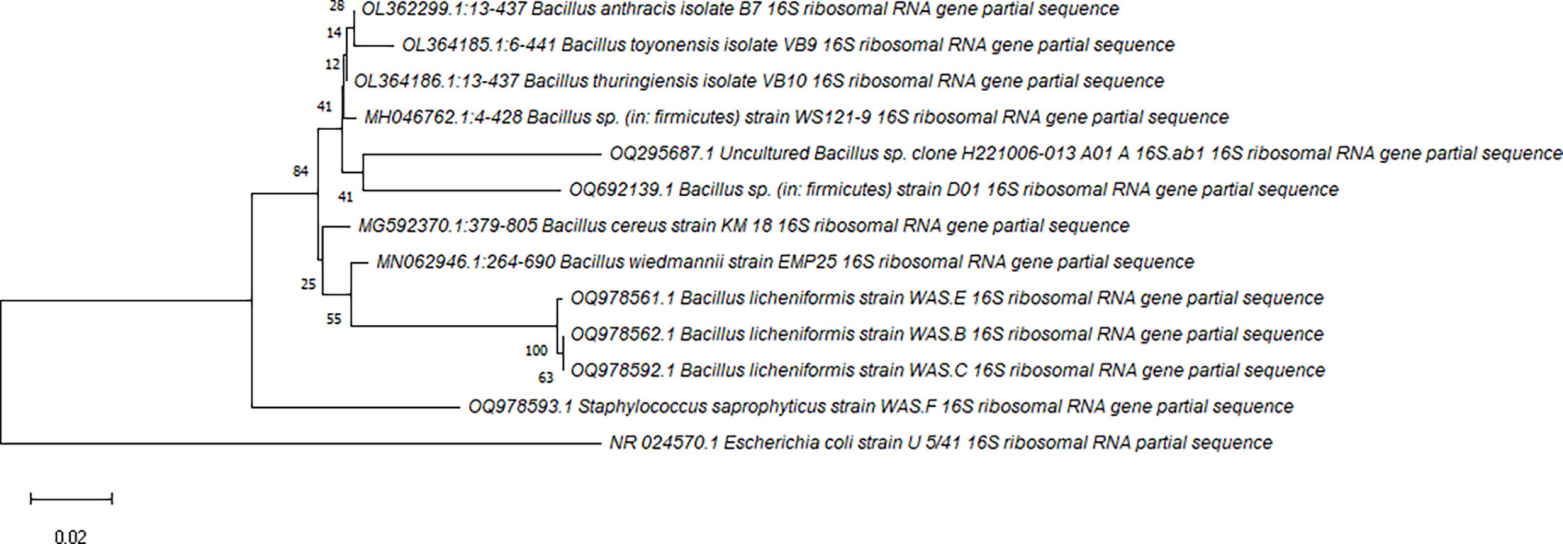
Phylogenetic tree based on the 16S rRNA sequence results of selected six isolates and six strains of *Bacillus* as ingroup and *E. coli* as outgroup using mega X tool.

Based on 16S rRNA sequencing, the phylogenetic tree (Fig. 2) highlights the relationship between the strains that have been characterized in this study. There are two clades originating from a common ancestor, one with the *Bacillus* sp. strains and the other with *S. saprophyticus*, with *E. coli* as outgroup. Within the two clades of *Bacillus* sp. strains, there are two monophyletic groups. One group shows that the two strains, US1-1 (OQ295687) and US2-2 (OQ6921391), from under-construction sites are sister groups. They share some characteristics with *B. anthracis*, *Bacillus toyonensis*, *Bacillus thuringiensis* and *Bacillus* sp. (in: *firmicutes*), whereas for the second group, the three strains, US1-2, US2-1 and MS1-1, make sister group based on their identity as *B. licheniformis*.

## Discussion

Khewra Salt Mine being the oldest mountain range in Pakistan has coloured mineral-rich salt contained in salt rocks, tunnels and lakes [27]. Khewra offers salt in various colours, ranging from transparent to pink and reddish. Having its own flora and fauna, this salt mine has many diverse bacterial communities. Six halophilic microbes have been identified by Haroon *et al*. [14], such as *Bacillus tequilensis*, *Bacillus xiamenensis*, *B. megaterium*, *S. pasteuri*, *P. aeruginosa* and *P. putida*.

**Fig. 3.**
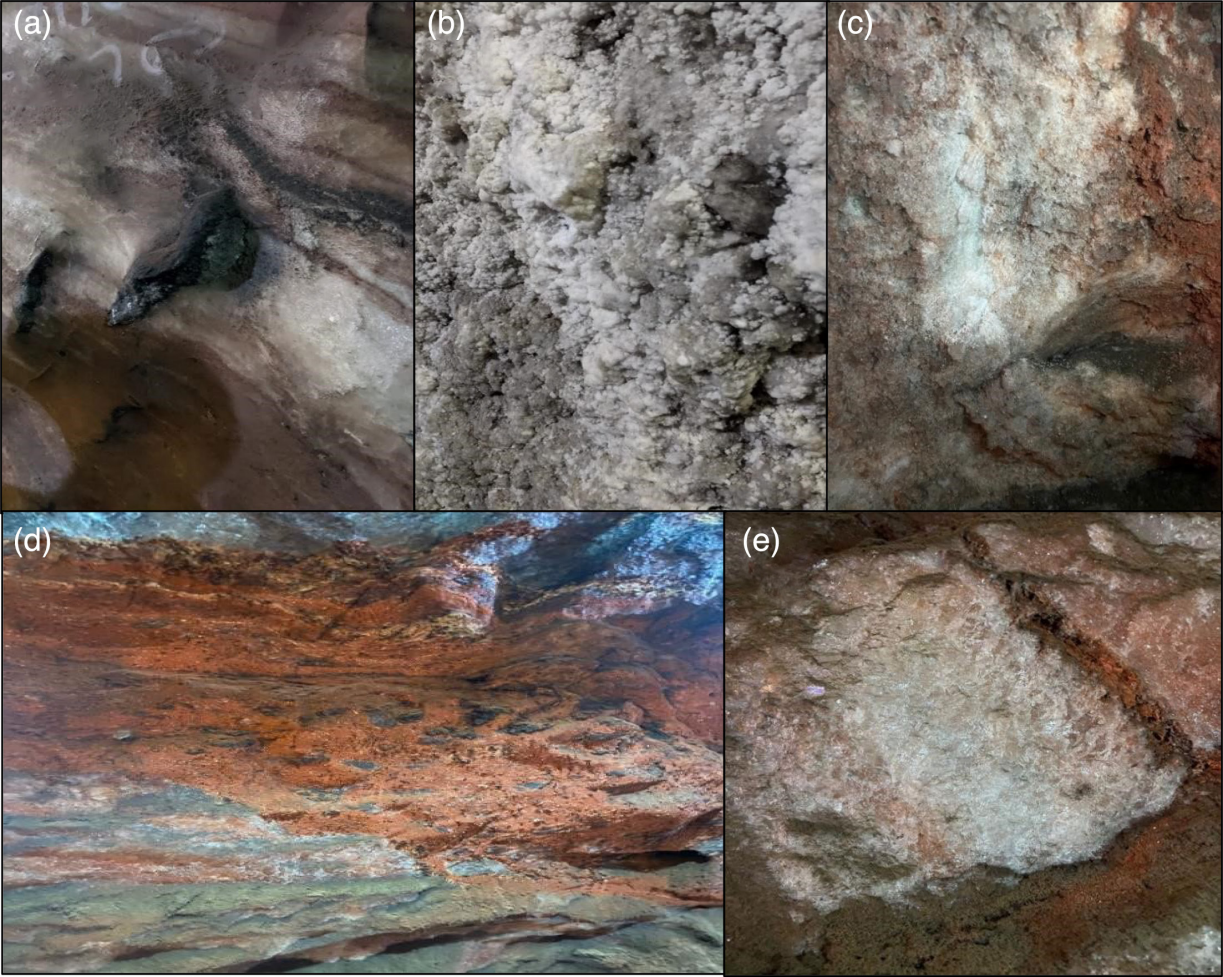
From top left to right: entrance salt wall (ES) (a), Chandni Chowk wall (CS) (b) and museum salt wall 1 (MS-1) (c). From bottom left to right: museum salt wall 2 (MS-2) (d) and museum salt wall 3 (MS-3) (e).

The current study pursues to explore bacterial diversity within Khewra Salt Mine in the hope of identifying bacterial species in the salt walls as opposed to previous research in saline water, soil and halophytes. Salt was taken from different points along the mine, as shown in Figs3  4 and abbreviated as ES, CS, MS-1, MS-2, MS-3, US-1 and US-2. The physical properties of salt, including colour, texture and pH as shown in Table 2, revealed that there are a variety of salts available in Khewra. The pH of 7.02–7.03 was indicative of salts dissolving in water and making a neutral solution. The bacterial cultures were grown from these salt samples on nutrient agar plates. Seven salt samples retained 20 bacterial colonies, which were then cultured on 2% NaCl nutrient agar to screen halophiles which eliminated ES-1 that showed no growth. The growth of bacterial strains on 2% NaCl is conclusive of slightly halophilic strains that can tolerate less than 5% NaCl concentration [7]. Further colony morphology, cell morphology and biochemical and molecular tests were done on the remaining 19 halophilic strains.

**Fig. 4.**
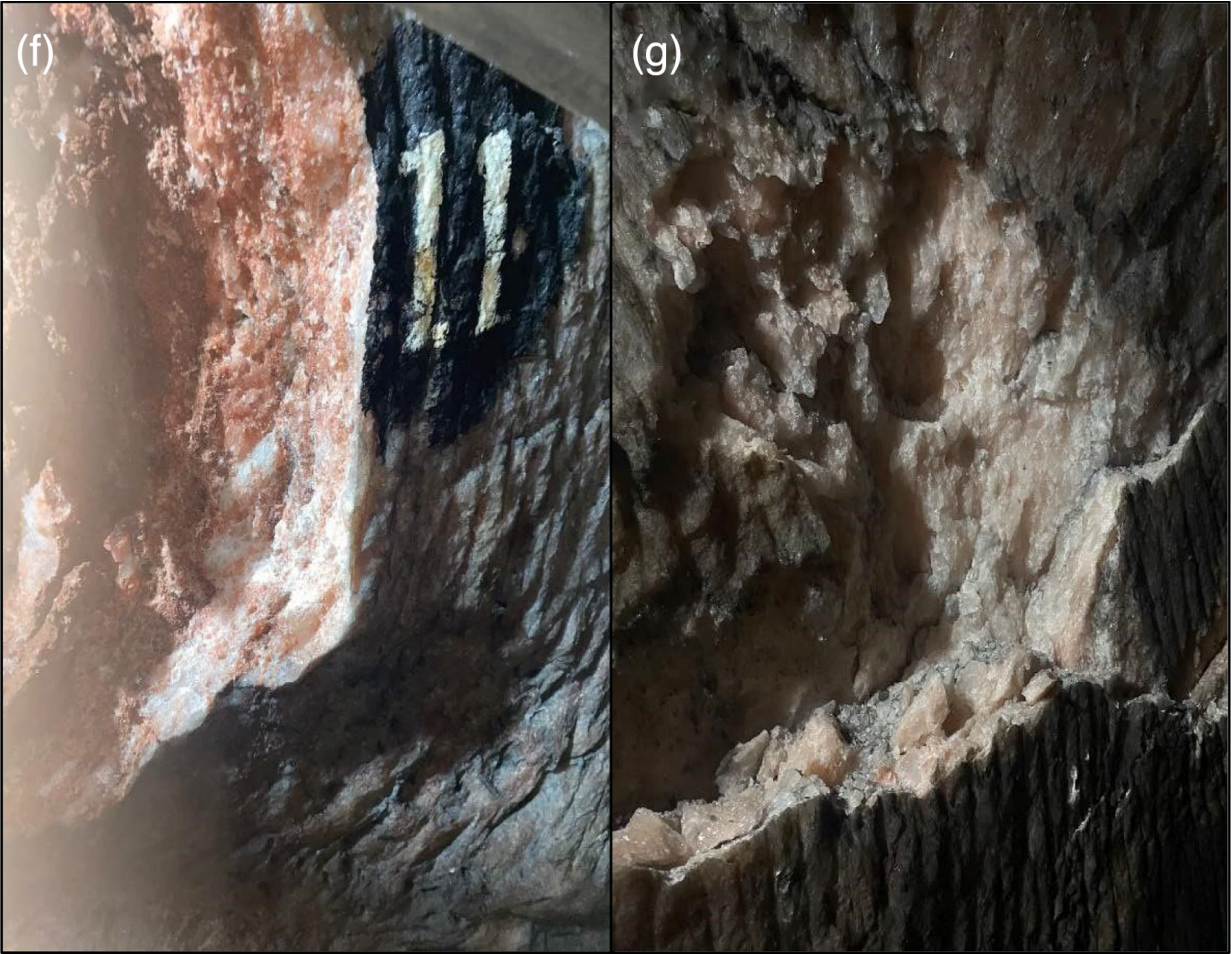
Under-construction part of Khewra Mine where two different salt variations are visible; on the left is US-1 (f), and on the right is US-2 (g).

The colour of colonies was predominantly creamy white, and a few were yellow and orange. The variation in pigmentation of colonies colour is due to the carotenoid pigments found in the cell membrane of halophiles [28]. Some colonies like ES-2, ES-4 and US2-1 appeared filamentous with wrinkled surface. Most of the colonies were round with entire margins. When observed for opacity, only CS-1, MS2-1, US1-1 and US1-2 were translucent, and the remaining were opaque. These colony morphologies of halophilic bacteria follow the description for *Halobacteriales* in a manual provided by Oren *et al.*[29].

Gram staining analysed the cellular morphology of isolates among which only two appeared Gram-negative as listed in Table 3. Most of the isolates proved to be Gram-positive cocci suggestive of *Staphylococcus* and *Streptococcus* species, and some appeared Gram-positive rods suggestive of *Bacillus* species. Biochemical tests characterized the bacterial isolates through catalase, oxidase and carbohydrate fermentation (Table 4). Catalase tests detect the enzyme catalase that actively converts hydrogen peroxide into water and oxygen. Among the isolates, all showed catalase activity except CS-2. The oxidase test detects the enzyme cytochrome oxidase, and the majority of the isolates appeared oxidase positive. Carbohydrate fermentation determines the ability of bacteria to ferment sugars such as glucose and sucrose as a source of energy for which all were positive.

Upon careful consideration of their biochemical activity, colony morphology and sample location, US1-1, US1-2, US2-1, US2-2, MS1-1 and MS1-2 were selected for biochemical tests of SIM and citrate utilization (Table 5). SIM detects sulphur reduction, indole production and motility. All isolates showed motility except MS1-1. All isolates turned positive for indole production and negative for sulfide. The citrate test determines whether bacteria can utilize citrate as a source of energy, for which three were positive and three negative.

For the assessment of salt concentrations needed for growth [29], bacterial isolates were further subjected to 2, 4 and 6% NaCl concentrations. The pure isolates only grew on 2 and 4% NaCl concentrations, which proves that they are slightly halophilic requiring less than 5% NaCl concentration as depicted in Table 6 [7]. The next critical step was to identify antibiotic sensitivity among the isolates. Antibiotics discs of penicillin (10 µg ml^−1^), ampicillin (10 µg ml^−1^), chloramphenicol (30 µg ml^−1^) and erythromycin (15 µg ml^−1^) were used. All isolates were sensitive to chloramphenicol and resistant to ampicillin except US1-1, which appeared to have a 20-mm zone of inhibition in response to ampicillin (Table 7). Antibiotic resistance is crucial for their survival and can be attributed to genetic mutations resulting from horizontal or vertical gene transfer in bacteria.

The statistical analysis of the antibiotic test results was performed using the ANOVA calculator (AAT Bioquest). The one-way ANOVA (Table S1) and the *post-hoc* tests (Table S2) yielded *P*-value <0.00001, which accounts for significant differences in the antibiotic susceptibility of strains. The *P*-values for rythromycin, penicillin and chloramphenicol were found to be <0.05, showing significant differences in reference to the control group and high susceptibility to the antibiotic. However, the tested strains were found highly resistant to ampicillin showing no significant difference. As for the pairwise *t*-test (Table S3), some of the antibiotic pairs showed significant variation, while others like erythromycin vs. penicillin showed no significant differences in susceptibility.

The phylogenetic tree depicts the relationship of bacterial strains based on 16S rRNA sequencing. The isolate MS1-2 originates from a common ancestor of *E. coli* strain, with 100% similarity to *S. saprophyticus* strain CJJH1. *S. saprophyticus* has also been found in saline samples of Khewra Salt Mine by Sarwar *et al.* (2014) [16] and soil samples of halophyte rhizospheres by Mukhtar *et al.* [15]. The isolated strains of the under-construction area, US1-1 and US2-2, belong to sister clades, each with a 94% similarity to uncultured *Bacillus* sp. and *Bacillus* sp. PSA3, respectively. The discovery of *Bacillus* strains from our salt samples corresponds to the previous findings of *Bacillus* species by Akhtar *et al.* [24] and Haroon *et al.* [14]. Closely related species of *firmicutes* have also been discovered by Roohi *et al.* [7] and Mukhtar *et al.* [15] in Khewra Salt Mine. The isolates US1-1 and US2-2 appear as Gram-positive rods, forming creamy white or yellow colonies on nutrient agar in accordance with *Bacillus* species [30]. They test positive for catalase and oxidase whereas negative for citrate utilization.

Among others, isolates US1-2, US2-1 and MS1-1 emerge from a sister clade with *Bacillus wiedmanni,* exhibiting 100% similarity to *B. licheniformis* strain P8. Another research in [24] has also revealed strains of *B. licheniformis* from soil and water samples. These isolates correspond to the characteristics of *B. licheniformis* being Gram-positive rods that utilize citrate and possess catalase and oxidase activity [31]. However, US2-1 appears to be coccobacilli rather than rods. This morphological feature suggests evolutionary changes in its genetic material.

The current findings are backed by previously conducted research on halophilic bacteria inhabiting the rhizosphere of halophytes, aimed at inducing salt tolerance in wheat, discovered a few isolates as *B. tequilensis, Bacillus xiamenesis* and *B. megaterium* [14].

*Bacillus* species, known for their genomic diversity, inhabit a wide variety of ecological niches [32]. The recovery of *Bacillus* strains from the under-construction region in Khewra validates how survival instinct makes them a commonly harvested organism with potential applications in industry, medicine and agriculture. The strain of *S. saprophyticus* discovered in MS1-2 may have emerged from tourist contamination as the species itself is an opportunistic pathogen well-known for causing urinary tract infections. The discovered strain of *B. licheniformis* derived from US1-2 and US2-1, under-construction area, seems to have evolved to tolerate salt stress and hold significant potential to induce salt tolerance in plants. *B. licheniformis* has widespread applications in medicine, as a probiotic to prevent pathogen colonization in the gut [33], and its strain DW2 for industrial manufacture of bacitracin, a topical antibiotic ointment [34]. In agriculture, it is utilized as a biostimulant to enhance plant growth in drought stress [35] and an active fungicide [36].

Halophilic bacteria have diverse applications including the extraction of antimicrobial agents and the production of enzymes such as proteases, amylases and lipases [37,39]. *Vibrio azureus* has antifungal action against *Candida albicans* [40], and *P. aeruginosa* has antibacterial impact on *Staphylococcus aureus* [41]. Bioremediation is another approach towards a sustainable environment through the synthesis of phytohormones in halophilic bacteria [42]. Halophiles have been extensively studied for agricultural applications such as inducing salt-tolerant genes in crops like wheat [14].

## Conclusion

This study was able to explore the diverse microbiome of halophiles present in the salt walls of the Khewra Salt Mine. Through 16S rRNA sequencing, the selected 6 isolates among 19 halophiles obtained from 7 salt samples, including 2 novel strains from the under-construction area of the mine, were categorized as slightly halophilic requiring 2–4% NaCl. The phylogenetic analysis revealed two distinct monophyletic groups: one containing strain related to *B. anthracis*, *B. toyonensis* and *B. thuringiensis* and the other consisting of strains all identified as *B. licheniformis*. These halophilic bacterial isolates portray potential applications in genetic engineering for combating drought and salinity in agriculture [14], as well as for industrial enzyme production due to their salt-tolerant genes [15]. This research provides valuable insights into the microbial ecosystems thriving in extreme environments such as salt mines, emphasizing the importance of studying these unique habitats for biotechnological advancements.

## Supplementary material

10.1099/acmi.0.000869.v4 Uncited Supplementary Material 1.

## Abbreviations

CS: Chandni salt
ES: entrance salt
I: intermediate
MS: museum salt
NCBI: National Center for Biotechnology Information
NJ: neighbour-joining
PMDC: Pakistan Mineral Development Corporation
R: resistant
S: sensitive
SIM: sulfide indole motility
US: under-construction salt
