# Supplementary material for: 16S rRNA gene sequencing reveals bacterial diversity in Khewra Salt Mine walls
Source: Access Microbiol. 2024 Dec 18;6(12):000869.v4. doi: 10.1099/acmi.0.000869.v4 (PMC12453392; doi:10.1099/acmi.0.000869.v4)
Supplement: Uncited Supplementary Material 1. [file acmi-6-00869-s001.pdf]

16S rRNA Gene Sequencing Reveals Bacterial Diversity in Khewra Salt Mine Walls

Supplementary Data

Table S1: Summary of One-way ANOVA.

	Degrees of Freedom (DF)	Sum of Squares (SS)	Mean Square (MS)	*f*-Statistic	*p*-value
**Between Groups**	4	7704.5556	1926.1389	39.6666	1.0447e-18
**Within Groups**	85	4127.4444	48.5582		
**Total**	89	11832			

Table S2: Summary of Tukey's Multiple Comparison.

Group	Difference	Lower	Upper	*p*-value adjusted
**Control-Erythromycin**	19.7778	13.303	26.251	2.1357e-
**Control-Ampicillin**	4.3889	-2.0852	10.863	0.3309
**Control-Penicillin**	15.8333	9.3593	22.307	1.2851e-
**Control-**	24.4444	17.970	30.918	2.0838e-
**Erythromycin-Ampicillin**	-15.3889	-21.863	-8.9148	2.9972e-
**Erythromycin-Penicillin**	-3.9444	-	2.5296	0.4406
**Erythromycin-Chloramphenicol**	4.6667	-1.8074	11.140 7	0.2706
**Ampicillin -Penicillin**	11.4444	4.9704	17.918	0
**Ampicillin -**	20.0556	13.581	26.529	2.1136e-
**Penicillin -**	8.6111	2.137	15.085	0.0033

Table S3: Summary of Pairwise T-test (Pooled SD, Adjusted BH) Comparison.

	**Control**	**Erythromycin**	**Ampicillin**	**Penicillin**
**Erythromycin**	1.7281e-12	-	-	-
**Ampicillin**	0.0692	5.9879e-9	-	-
**Penicillin**	3.1763e-9	0.0931	0.0000067846	-
**Chloramphenicol**	4.5597e-16	0.0596	1.4852e-12	0.0005
